# Challenges in Orthopedic Surgical Decision-Making for Multilevel Vertebrae Fractures

**DOI:** 10.7759/cureus.68262

**Published:** 2024-08-31

**Authors:** Michelle O Uwefoh, Elizabeth Edah, Lee-Ann D Charles, Elvis Gini, Ekama Isong, Deborah A Ademo, Omavuaye J Eruvwedede, Daniel Ivankovich

**Affiliations:** 1 Orthopedic Surgery, All Saints University School of Medicine, Roseau, DMA; 2 General Surgery, All Saints University School of Medicine, Roseau, DMA; 3 Family Medicine, Windsor University School of Medicine, Cayon, KNA; 4 Internal Medicine, All Saints University School of Medicine, Roseau, DMA; 5 Pediatrics, All Saints University School of Medicine, Roseau, DMA; 6 Emergency Medicine, All Saints University School of Medicine, Roseau, DMA; 7 Orthopedic Traumatology and Adult Spine/Joint Reconstruction - Metro Orthopedics, OnePatient Global Health Initiative, Chicago, USA

**Keywords:** comorbidities, decision making, surgery, female, multilevel fractures

## Abstract

Vertebral fractures commonly occur in postmenopausal women due to decreased bone density, a condition known as osteoporosis. They can occur after minimal trauma or even during routine activities. Vertebral fractures occur predominantly in individuals with a high fall risk. This case report explores the clinical complexities surrounding a 65-year-old female patient with a history of multilevel vertebrae fractures compounded by a history of chronic smoking, osteoporosis, multiple falls, and evident signs of osteopenia on X-ray. These risk factors complicate the decision to perform surgery and highlight the importance of constantly weighing the benefits and possible risks. This paper aims to emphasize the gender-specific challenges healthcare providers encounter when assessing surgical risks in the context of postmenopausal females with significant comorbidities. It underlines the need for tailored and comprehensive care strategies to manage orthopedic conditions in high-risk female individuals, further aligning with one of the World Health Organization's concerns on addressing gender-specific health considerations.

## Introduction

Vertebral fractures are breaks that can occur anywhere on the spine and can result from trauma, metastasis, or degenerative conditions, most commonly osteoporosis [1]. This type of fracture can often be fatal and sometimes go undetected or underdiagnosed by clinicians and radiologists [1]. They can also cause significant loss of mobility and pulmonary difficulties [2,3]. Vertebral fractures usually indicate underlying bone health and relate to an arsenal of risk factors.

Surgical decision-making is a multifaceted process that ties in with a patient's medical history and lifestyle choices. Some barriers exist between orthopedic surgeons and primary care physicians regarding who can identify and treat osteoporosis, especially those with fragility fractures [4]. Vertebral fractures are commonly associated with postmenopausal osteoporosis and serve as a significant factor to consider in studies evaluating treatment options for said disease [1].

This paper explores the clinical case of a postmenopausal female with multilevel vertebral fractures. She has a past medical history of osteoporosis, which makes her vulnerable to fractures from recurrent falls. It highlights the challenges of treatment plans in patients of this demographic and how surgical decisions intersect with careful considerations of gender-based risk factors. We also highlight the forms of surgical management and the possible areas considered in the current literature that affect whether a patient undergoes surgery over conservative management.

In presenting this case, each element provides its own distinct level of significance. Osteopenia and osteoporosis introduce a myriad of bone health intricacies, where the fractures are not mainly isolated events but a sign of underlying illness. Because of the interactions between medical conditions and lifestyle choices, it is crucial to address the management of vertebral fractures while steering the complexities introduced by a patient's history.

## Case presentation

A 65-year-old Caucasian female with a past medical history of osteoporosis presented to the orthopedic surgery clinic in January 2022 with complaints of persistent postoperative left thigh and right ankle pain after a surgical procedure ten months prior due to multiple fractures of the left femur, tibia, and proximal phalanx of the right hallux following a fall. She also had a history of existing comorbidities like seizure disorder, anxiety, depression, hypertension, hypercholesterolemia, hypothyroidism, and a long-standing history of tobacco smoking for about 20 years.

**Figure 1:**
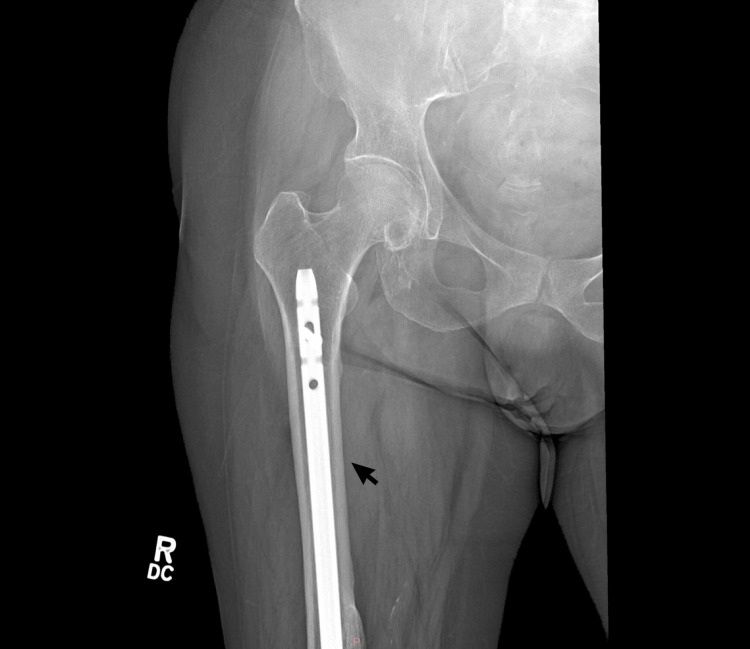
X-ray imaging showcasing healed fracture of the femur with hardware in-situ

**Figure 2:**
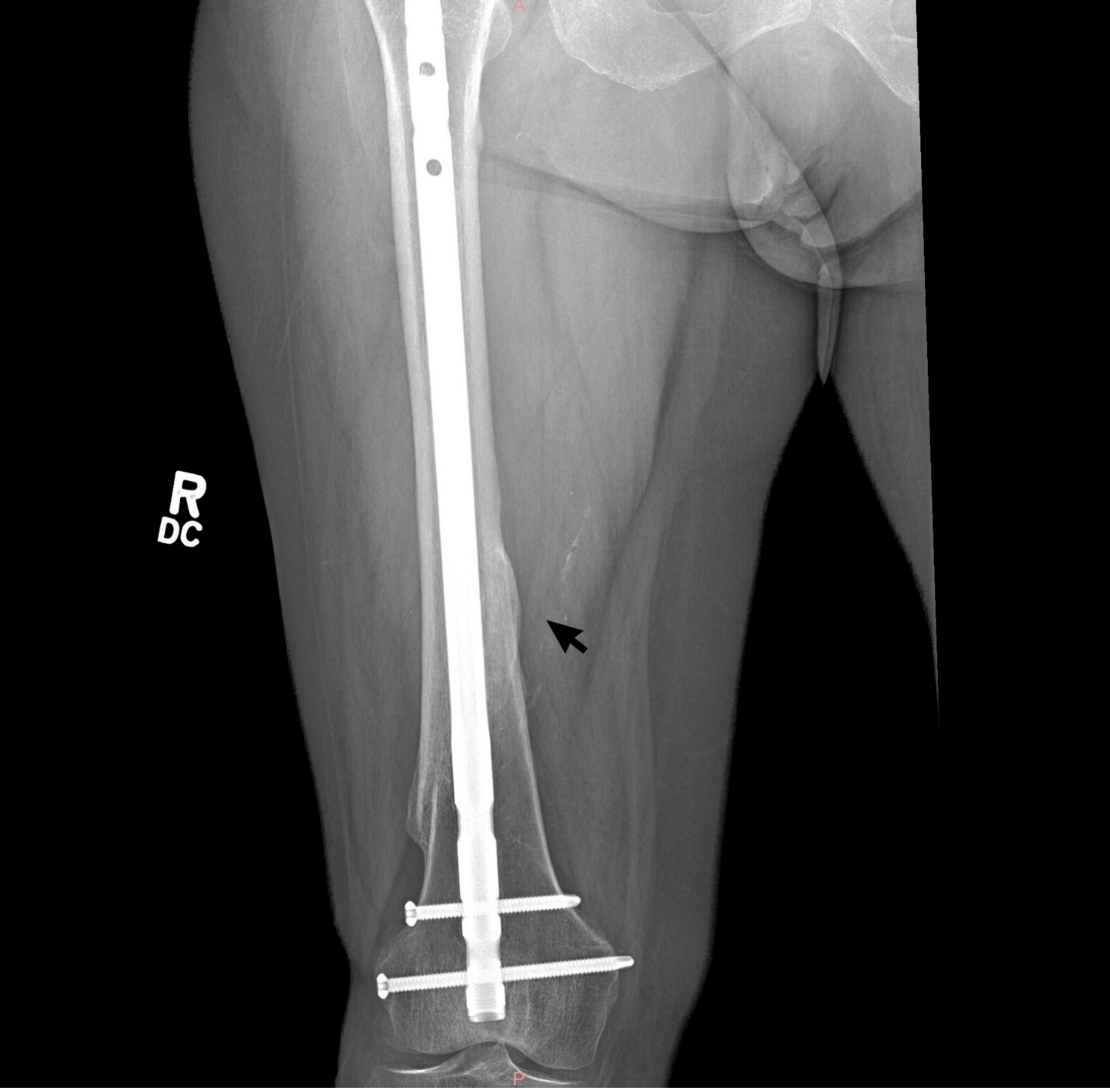
X-ray imaging showcasing healed fracture of the femur with hardware in-situ

**Figure 3:**
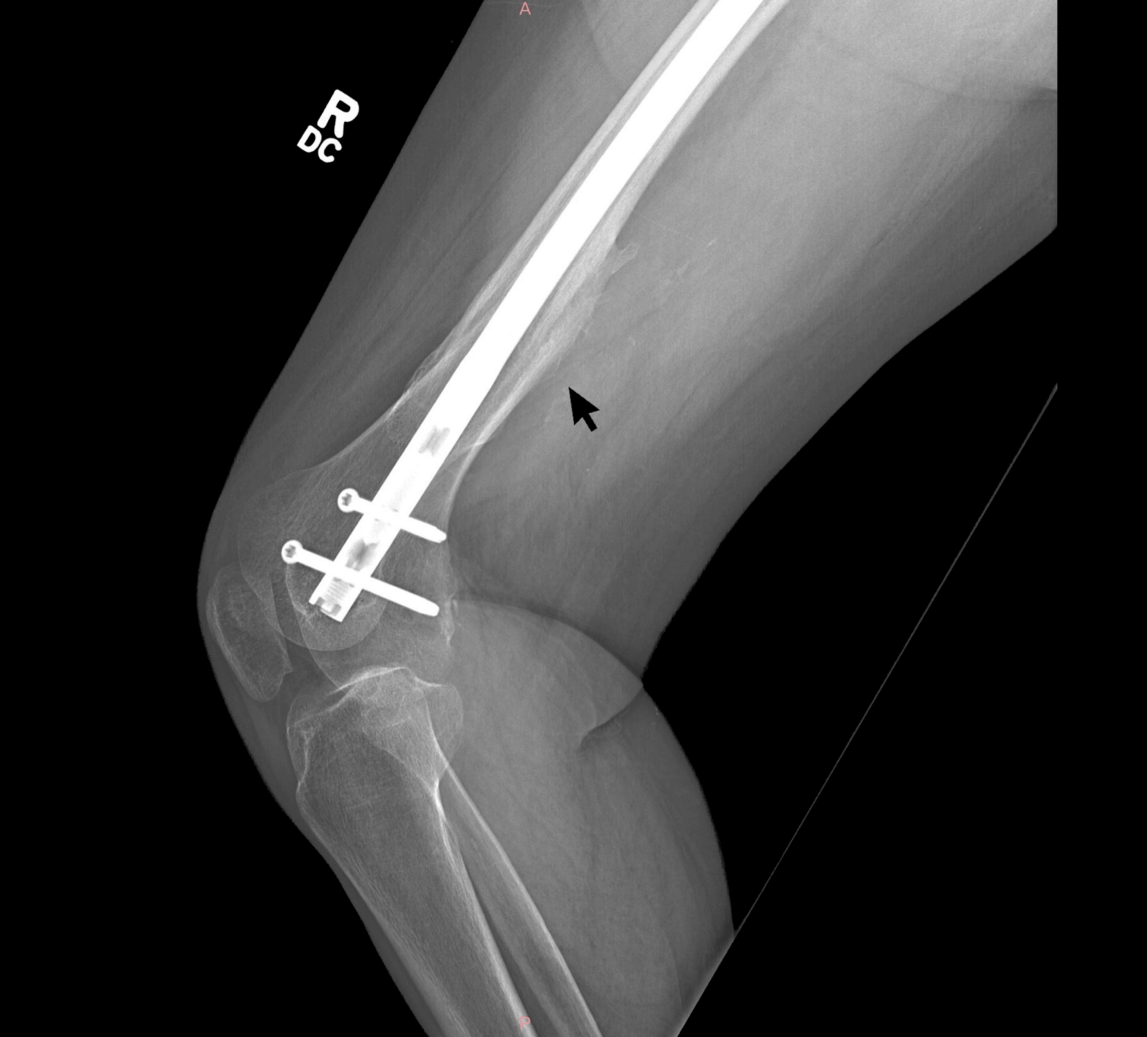
X-ray imaging showcasing healed fracture of the femur with hardware in-situ

During the physical examination, the patient presented with an antalgic gait requiring an assistive device for ambulation. There was mild to moderate swelling of the right knee with associated medial joint pain, crepitation on joint movement, and limitations to flexion and extension. The left knee demonstrated mild to moderate crepitation but no instability. There was also minimal left knee swelling and medial joint pain. Left ankle inspection did not identify any deformities or misalignment of bones. Upon review of X-ray imaging, the fractures were healed entirely (Figures 1-3). After discussions with the patient, it was decided that the removal of the hardware screws placed during her previous surgery would most likely minimize the pain and discomfort she experienced. The patient was also offered physical therapy, which she refused and cited previous trials of failed sessions as her reason. She believed it would be ineffective since she experienced no improvements previously. She was prescribed Tramadol 50 mg and asked to come in for a follow-up appointment at the clinic in four weeks. She was also counseled on smoking cessation and its benefits in improving her risk factors for subsequent fractures and healing.

**Figure 4:**
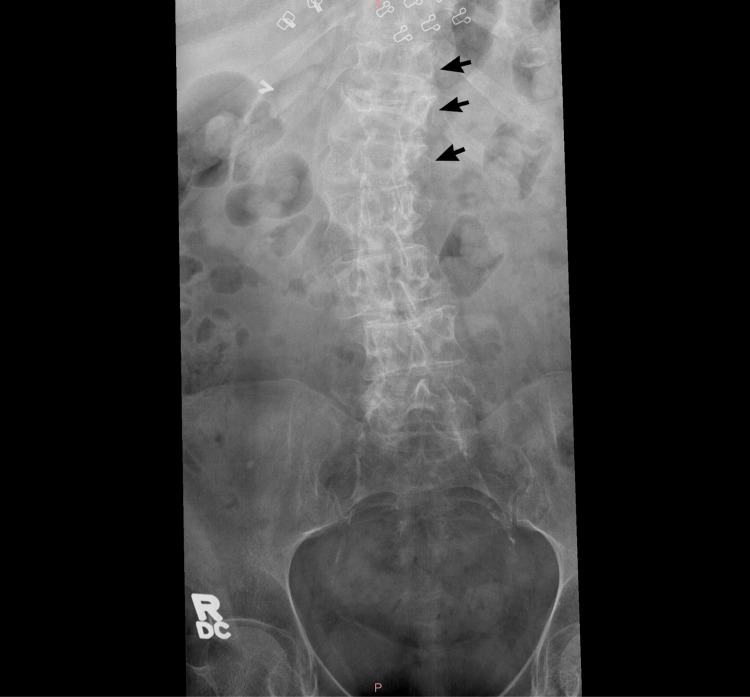
X-ray imaging showcasing compression fractures at T11, T12, and L1 levels, as well as degenerative scoliosis of the thoracolumbar spine

She returned to the clinic eight months later with continued complaints of persistent pain. Further imaging revealed that she was severely osteopenic. X-rays of the lumbar spine showed compression fractures at the levels of T11, T12, and L1 (Figure 4), as well as degenerative scoliosis of the spine, while lateral and AP views of the right femur and left tibia showed sufficiently healed fractures. The orthopedic surgeon reiterated to the patient that her pain was most likely from the screws in her femur but now also in combination with referred pain from her spine. She was also counseled on her risk factors, such as smoking and her postmenopausal status, which are contributors to her severe osteopenia resulting in the compression fractures identified.

Further discussions about the care plan occurred. The patient was debriefed on the multiple options of treatment, such as surgical hardware removal, intra-articular steroid injections, and physical therapy. The patient was advised to visit her primary care physician to manage her other comorbidities. She was also encouraged to return to the clinic one week after that visit to continue further treatment discussions.

**Figure 5:**
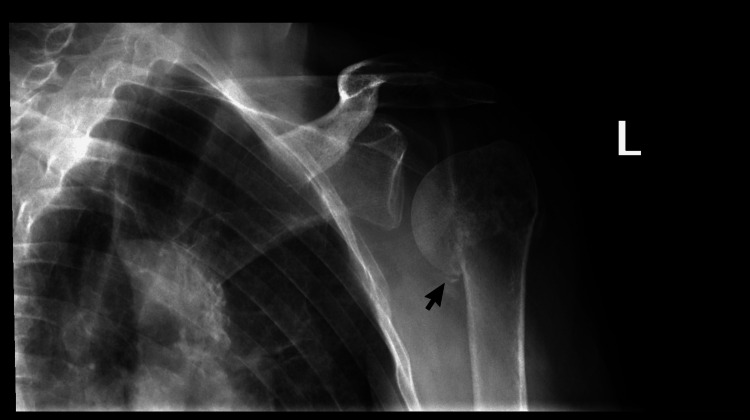
X-ray imaging showcasing fracture of the surgical neck of the left humerus

She eventually returned to the clinic six weeks later with new complaints of left shoulder pain due to a fall she had sustained three days prior. X-rays revealed a three-part fracture of the surgical neck of the left humerus (Figure 5). The patient was instructed to use an immobilized sling for three to four weeks and prescribed hydrocodone daily for seven days to manage her pain. She was scheduled for a follow-up appointment three weeks later, and upon her return during this visit, occupational and physical therapy were discussed. She was also given a prescription for a 30-day supply of hydrocodone 5 mg and acetaminophen 325 mg tablets for her pain. The patient was advised to follow up in four weeks; however, she returned six months later with continued complaints of left shoulder pain. AP and lateral X-ray views of the shoulder demonstrated complete healing of the fracture.

**Figure 6:**
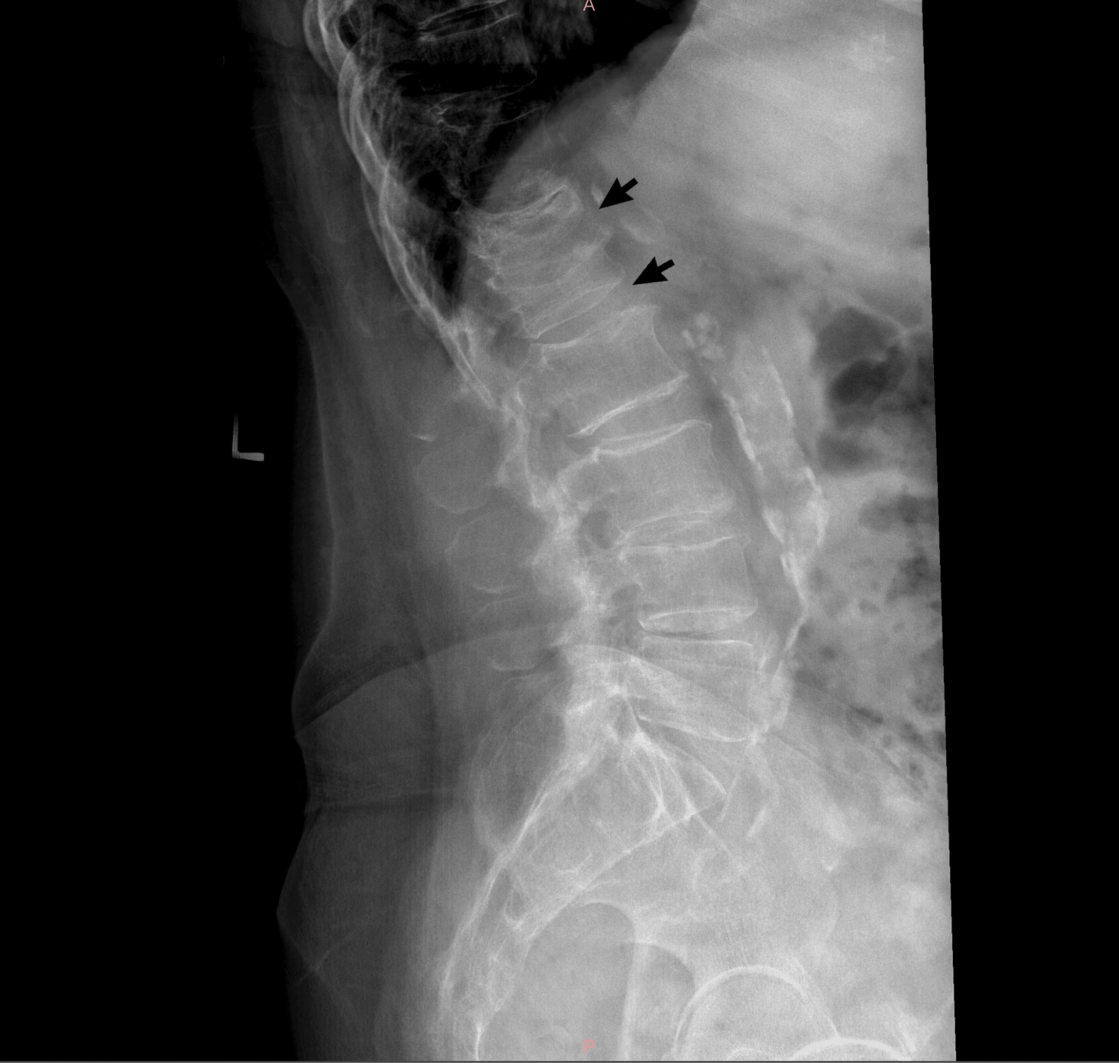
X-ray imaging showcasing degenerative scoliosis and stenosis at L3-L4 level, as well as an L1 fracture

**Figure 7:**
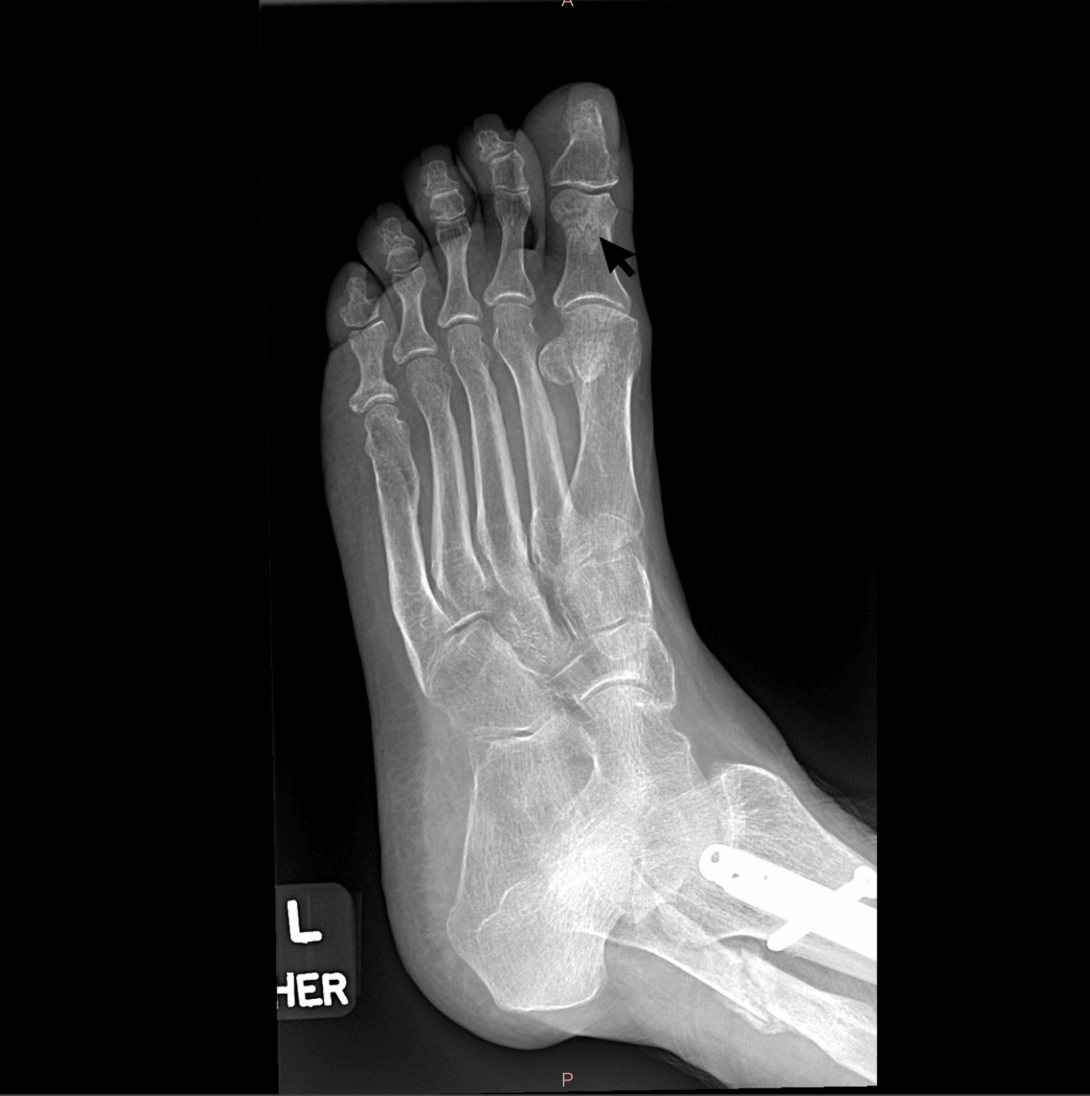
X-ray imaging showcasing fracture of the proximal phalanx of the left great toe

The patient was counseled on smoking cessation and advised to return to the clinic in two weeks. However, she only returned five months later with complaints of intense low back pain for two months. She was observed using a walker for ambulation and disclosed that she had recently slipped and fallen. The patient stated that she could not recall the exact incident date. A physical exam revealed tenderness of the lumbar spine and paraspinal muscles. Lumbosacral X-ray revealed degenerative scoliosis, significant stenosis at L3-L4 level with multiple foraminal stenosis noted, and an old L1 fracture with 70% loss of height and diffuse osteopenia/osteoporosis (Figure 6). X-ray of the left foot revealed a fracture of the proximal phalanx of the first toe (Figure 7). The patient was given a cast boot to aid in recovery. She was also advised to follow up with her primary care physician and was referred for neurosurgical evaluation. Additionally, they counseled her on the benefits of smoking cessation and scheduled her for a follow-up appointment in eight weeks, but she did not attend.

Upon return to the clinic three months later, the patient detailed an episode of another fall three weeks prior and subsequent right femur pain as a result of the fall. The patient's X-rays revealed no obvious deformity, prompting a referral for an MRI of the thoracic and lumbar spine, as well as to a pain specialist. The plan involved continuing conservative management, encouraging compliance with follow-up appointments, and encouraging smoking cessation, as her assessment consistently indicated that the risks of surgery outweighed the benefits.

## Discussion

The risk of developing vertebral fractures increases with increasing age [5]. The European Vertebral Osteoporosis Study (EVOS) reflects this increase in the overall prevalence of vertebral deformity with age in both genders. The risk in women increases from 5% at age 50 to 25% at age 75 and from 10% to 18% for men of the corresponding age group [6]. A cross-sectional study of 3330 US adults aged ≥40 by the National Health and Nutrition Examination Survey (NHANES) in 2013-2014 placed the overall prevalence of vertebral fractures in the US at 5.4% for both men and women [7]. Similar to other reports in the literature, the prevalence increased from <5% in those aged <60 to 11% in individuals aged 70-79 and 18% in those ≥80 years of age [7]. The Rotterdam study retrospectively observed 3469 men and women. The findings revealed an absolute increased incidence of vertebral fractures in women, even with an already increased incidence with age in both genders [8]. Studies have cited men having an overall higher peak bone mineral density than women as a significant reason for this difference. Another reason cited is the higher rate of bone loss experienced by women due to age-related changes [9,10]. 

When the load placed on a bone outweighs its strength, it can lead to a fracture, and the ability to resist this load depends on the bone's volumetric mineral density and cross-sectional area. Even though men have more giant bones, they also have bigger muscles, greater body height, and body weight [11]. All these ensure that the load imposed per unit cross-sectional area on the vertebral body is the same in men and women. With increasing age, vertebral bodies become less appropriately adapted, increasing the risk of fractures [11]. Our patient's extensive smoking history is another variable to consider; research estimates that smoking can increase the lifetime risk of developing a vertebral fracture in women by 13% [12]. Our patient's age and extensive smoking history, alongside hormonal and age-related changes, explain her high susceptibility to multilevel vertebral fractures. 

About three in four vertebral fractures frequently go clinically unrecognized due to a lack of symptoms or difficulty in pinpointing a cause of symptoms when present. The most common presenting complaint is back pain, but not all cases of back pain are initially suspected to be related to vertebral fractures. Therefore, physicians can sometimes miss it during clinical evaluations [13,14]. Imaging techniques are the gold standard for diagnosing vertebral fractures. Plain radiographs, computed tomography (CT), and magnetic resonance imaging (MRI) can all be utilized. Still, plain radiographs are the initial diagnostic modality, while CT is considered the best modality for further evaluation of spinal fractures [15]. In a large randomized cohort study, it was observed that in older patients of any gender who had a chest radiograph taken for any reason in the emergency department during one year, there was a 16% prevalence of moderate-to-severe vertebral fractures [16]. However, 40% of these fractures were absent in the official radiologists' reports [16]. Another retrospective study of 934 women 60 years and older observed that 14% of these women had radiologic evidence of moderate or severe vertebral fractures, and only 50% of these fractures were included in the radiologist's reports [17]. Similar reports from various studies suggest a worldwide problem in detecting and diagnosing vertebral fractures, and possible reasons across the board are a failure to point out or interpret findings on radiographs by radiologists, the lack of a generally adopted standardized protocol in identifying vertebral fractures, and sometimes ambiguity in the description of the radiographic findings even when mentioned [13,18].

Another objective moving forward, even after a diagnosis, is preventing future fractures, as the risk of subsequent vertebral fractures increases after a single episode [19]. A study on osteoporosis-associated vertebral fractures with 694 patients highlighted that the living conditions of most patients in the age group frequently affected could be either residing in a care home, living on their own, or with family. 24% of patients affected in this study required professional domestic help, especially patients 70 years and older. When patient gender differences were considered, statistical analysis revealed that the requirement for domestic help appeared significantly more often in women than in men. A notable finding was that the male patients had significant domestic aid from their female life partners and not the other way around. Like our patient who lives alone without any domestic help, female patients in that category and age group considered in the study might be at an even higher risk of developing more fractures after their first presentation.

In the same study of 694 patients, when considering gender in prevention strategies with patients who had a definite diagnosis of osteoporosis like ours, statistics reveal that women were significantly seen to be on primary or secondary methods of prevention than men [20]. This finding highlights the advantage of early detection of vertebral fractures and establishing a definitive diagnosis when investigating possible causes of these fractures.

In patients with osteoporosis or at risk for osteoporosis, low-impact trauma or falls are common precursors of vertebral fractures, leading to different decisions being made concerning management [15]. As individuals age, the risk of falls increases, and certain factors like a previous fall, vestibulopathies, impaired vision, impaired balance/mobility, and decreased muscle strength also further increase this risk [21]. Patients with osteoporosis have been reported in the literature to experience impaired balance and mobility, as well as reduced muscle strength due to a reduction in range of motion and deformities that occur [21,22]. The impact of a fall is said to be worse in patients with osteoporosis and results in multiple axial and appendicular skeleton fractures [22]. Studies have also shown that fall prevention strategies contribute immensely to improving bone health in these patients. These strategies include but are not limited to fall-proofing homes by installing grab bars, encouraging weight-bearing and muscle-strengthening exercises, and monitoring medications for side effects like dizziness [22].

After a vertebral fracture, some patients require conservative management, which includes bed rest, physical therapy, analgesics, bisphosphonates, and bracing to enhance patient comfort [15]. Treatment of the underlying cause is also highly recommended, and surgery is considered in certain severe cases of pain or in an event where conservative management fails [15]. The most common surgical procedures for managing vertebral fractures are vertebroplasty and kyphoplasty [15,23]. Vertebroplasty involves injecting a needle with cement into the vertebral body under guided imaging. The cement hardens fast and can stabilize the fracture [15,23]. Kyphoplasty is a similar procedure, but the difference is utilizing a balloon to expand the vertebral body before the cement is injected [15,23]. The advantages of both procedures include the ability to treat multiple vertebral fractures simultaneously and the ability to complete them under sedation [23,24]. When comparing both techniques, kyphoplasty costs more but appears to restore the fractured vertebra's height better and reduce the risk of serious complications [24]. There is controversial evidence regarding vertebroplasty in the current literature. Still, some suggest that carefully selecting patients who are candidates for surgical management of vertebral fractures is the key to achieving success [24].

In a study describing demographic and economic trends in patients who had surgery for vertebral fractures, it was found that most surgeries are performed on males (59%), with most patients being between the ages of 18 and 44 (40%) [25]. A possible speculation for this finding could be the prevalence of low bone mass in older adults, especially females, as osteoporosis and low bone mass are essential to consider before making orthopedic surgical decisions [26]. Studies show that many patients with osteoporosis develop new vertebral fractures post-vertebroplasty [27,28]. The decision to conservatively treat our patient was based on the integrity of her bones, which, coupled with her social and past medical history, makes her a high-risk surgical patient and decreases her chances of postoperative success.

The decision to undergo surgery for any patient is preceded by considering many factors and discussing risks and outcomes in plain terms with the patient. Patients with significant pre-existing comorbidities, such as our patients, usually require pre-surgical considerations. A study published in 2022, looking into the decision-making process for a different orthopedic procedure, total knee replacement, revealed that women had less knowledge of procedures and conditions pre-discussion [29]. The differences observed were not explained by educational quotas [29]. This suggests that adequate education of patients, especially females, can be fundamental in improving a patient's cooperation during medical decision-making. Gender bias, albeit unconscious, can affect surgical decision-making in general, with reports showing inadequate communication methods when patients are female; underestimation of female pain levels by healthcare professionals is also well-documented in the literature [29]. Also, compared to men, females have been observed to shy away from procedures with long recovery times, as this can impede their performance of daily activities and impact caregiving roles [20,29].

In a systematic review of sex or gender determinants following spinal fusion surgery, 36 of the studies analyzed reported gender-related differences in postoperative outcomes, while the remaining described equivalent results. Of the 35 studies, 21 were on degenerative diseases, five were on spinal deformities, 3 were on spinal fractures, and 7 considered degenerative conditions and deformities together. One of the studies on spinal fractures following spinal fusion surgery observed postoperative fractures more commonly in females, patients with advanced age, and obese patients, while another observed males having better outcomes than females after thoracolumbar burst fractures [30]. In a retrospective study in 2021, 156 patients diagnosed with adult spinal deformity who had undergone spinal surgery were assembled and stratified to investigate if, in terms of gender, there was any significant difference in postoperative complication rates and quality of life [31]. The mean age of the patients included was 69.53, and no difference concerning the sex of the patient's sex was observed in this study [31]. However, a retrospective cohort study done in 2019 reflected that outcomes of surgery for cervical spinal fractures were much better in women compared to men [32]. It is our observation that women underwent surgery less often, but men had a higher one-year mortality [32]. While the current literature disagrees slightly with the existence of definitive differences between both sexes in terms of complications of spinal surgery, it does suggest that factors like comorbidities and unhealthy lifestyle practices tend to significantly affect the odds of certain complications like surgical site infections and postoperative mortality in general [30,33]. A huge risk factor for postoperative complications present in our case is smoking, as our patient had three failed cessation attempts. Studies have highlighted the benefits of smoking cessation before surgery in the reduction of post-surgical complications and the production of beneficial outcomes like decreasing the rate of postoperative wound infections [34,35].

Our case highlights the importance of carefully considering surgical risks and benefits in patients of these demographics and how much orthopedic surgeons can benefit from being aware of their specific challenges. It is highly encouraged to examine patients individually before making surgical decisions. Still, we believe the knowledge of these challenges aids physicians in making comprehensive decisions, especially in cases like ours. Data specifically looking at outcomes of surgery for multilevel vertebral fractures between men and women are scarce, and this is an area to highlight for possible future research. Other areas to consider are the comparison of risk factors and their implications on repeated or multilevel fractures in males versus females and any effects of socioeconomic differences between genders and how this impacts the risk for multilevel vertebral fractures, orthopedic surgery decision-making, and post-surgical outcomes.

## Conclusions

The case of a 68-year-old Caucasian female with multilevel vertebral fractures sheds light on the complex relationship between gender-specific concerns and orthopedic surgical decisions. There are significant health concerns linked to osteoporosis amongst older patients due to the increased chance of fractures in this population. Our patient’s past medical history emphasizes the importance of considering comorbidities, social supports, and lifestyle before making a decision to undergo surgery. With the patient’s risk factors and increased chance of surgical complications, conservative therapy was chosen over surgical interventions like kyphoplasty and vertebroplasty. Improving long-term quality of life will require modifications in risk factors like smoking cessation and fall prevention. When treating vertebral fractures, it is essential to take into account gender-specific risk factors and nuances, especially in late middle-aged patients. Finally, more research on the gender differences that exist and how closing these gaps can aid the successful management of patients with multilevel vertebral fractures is also highly encouraged.
